# Amlodipine rescues advanced iron overload cardiomyopathy in hemojuvelin knockout murine model: Clinical implications

**DOI:** 10.3389/fcvm.2023.1129349

**Published:** 2023-04-21

**Authors:** Pavel Zhabyeyev, Chandu Sadasivan, Saumya Shah, Faqi Wang, Gavin Y. Oudit

**Affiliations:** ^1^ Division of Cardiology, Department of Medicine, University of Alberta, Edmonton, AB, Canada; ^2^ Mazankowski Alberta Heart Institute, University of Alberta, Edmonton, AB, Canada

**Keywords:** iron overload, cardiomyopathy, hemochromatosis, calcium channel blockers, hemojuvelin

## Abstract

**Background:**

Iron overload cardiomyopathy (IOC) is a major co-morbidity of genetic hemochromatosis and secondary iron overload with limited therapeutic options. We aim to investigate mechanisms of rescue action of amlodipine in the murine model of iron overload, characterize changes in human cardiac tissue due to IOC, and compare them to the changes in the animal model of IOC.

**Methods and results:**

As an animal model, we used male hemojuvelin knockout (HJVKO) mice, which lacked hemojuvelin (a co-receptor protein for hepcidin expression). The mice were fed a high-iron diet from 4 weeks to 1 year of age. As a rescue, iron-fed mice received the Ca^2+^ channel blocker, amlodipine, from 9 to 12 months. Iron overload resulted in systolic and diastolic dysfunctions and changes in the cardiac tissue similar to the changes in the explanted human heart with IOC. An IOC patient (β-thalassemia) with left-ventricular ejection fraction (LVEF) 25% underwent heart transplantation. The murine model and the explanted heart showed intra-myocyte iron deposition, fibrosis, hypertrophy, oxidative stress, remodeling of Ca^2+^ cycling proteins, and metabolic kinases typical of heart failure. Single-myocyte contractility and Ca^2+^ release were diminished in the murine model. The amlodipine-treated group exhibited normalization of cellular function and reversed fibrosis, hypertrophy, oxidative stress, and metabolic remodeling. We also report a clinical case of primary hemochromatosis successfully treated with amlodipine.

**Conclusions:**

The aged HJVKO murine model on the iron-rich diet reproduced many features of the human case of IOC. The use of amlodipine in the murine model and clinical case reversed IOC remodeling, demonstrating that amlodipine is effective adjuvant therapy for IOC.

## 1. Introduction

Iron is an essential trace element that plays a crucial role in oxygen transport, oxidative phosphorylation, and the production of reactive oxygen species (ROS) (1, 2). Iron homeostasis is achieved by a concerted action of multiple proteins, whose actions converge on the SMAD signaling pathway controlling the expression of the *Hamp* gene encoding a suppressor of iron uptake, hepcidin (2–5). The two major regulatory proteins involved in the control of hepcidin expression are human hemochromatosis protein (HFE protein) and hemojuvelin (HJV) (4, 5). Mutations in these genes lead to primary hemochromatosis (iron overload) (6). Mutations in the *Hfe* gene are the most prevalent, constituting approximately 90% of adult hemochromatosis phenotypes in white populations of European descent (4–8). Mutations in *Hjv* (*Hfe2*) gene are considerably less frequent but lead to juvenile hemochromatosis with a high degree of iron overload and overt cardiac phenotype (7, 9). Secondary hemochromatosis primarily arises as a side effect of frequent blood transfusions used to treat congenital anemias (2, 10) and intravenous iron supplementation in hemodialysis (11). In the case of primary hemochromatosis, the current approach to treatment is an early screening of susceptible populations, so early interventions (dietary management and phlebotomy) can be used to control iron levels (4, 6). Secondary hemochromatosis is managed by chelation therapy to reduce iron cardiotoxicity (6, 12). Combining chelation therapy with antioxidants was also suggested [for review, see Wongjaikam, Kumfu (13)].

Another route for improving chelation therapy is to combine iron chelators with L-type Ca^2+^ channel blockers. Ca^2+^ channels aid iron cardiotoxicity by providing a major entry route for Fe^2+^ ions into cardiomyocytes (14–16). Clinical trials to investigate the effectiveness of Ca^2+^ channel blockers as an adjunct therapy to iron chelators have been started (17, 18), and one has been completed (19). Based on subgroup analyses, the AmloThal clinical trial (NCT01395199) concluded that amlodipine combined with chelation therapy reduced cardiac iron more effectively than chelation therapy alone (19). In light of these findings, mechanistic studies on the animal models are important to elucidate the mechanism of action of amlodipine in the settings of iron overload. Amlodipine is a dihydropyridine Ca^2+^ channel blocker mainly used to treat hypertension (20). Besides channel-blocking, the compound has antioxidant (21) and inhibitory activity on several enzymes at concentrations above 5 µmol/L (22, 23). The typical plasma concentration of amlodipine in patients is 10–20 nmol/L (24, 25), and in the mouse model of iron overload is about 650 nmol/L (15).

In this study, we report two cases of hemochromatosis (primary and secondary) managed by the L-type Ca^2+^ channel blocker (amlodipine) and compare them to the animal model of iron overload treated with amlodipine. In the case of secondary hemochromatosis, we obtained explanted tissue samples of the heart with iron overload cardiomyopathy (IOC). We compared these samples with non-failing controls to identify IOC-related changes. Then, we compared these changes to the analogous changes in the animal model of iron overload. As the animal model of iron overload, we used a 12-month hemojuvelin knockout (*Hjv*^−/−^) on a high-iron diet. That model has a more severe and pronounced phenotype resembling human IOC more closely than wild-type mice on a high-iron diet (26, 27). We also investigated the ability of amlodipine (i) to lower cardiac iron and (ii) to reverse maladaptive cardiac remodeling by introducing amlodipine at the age of 9 months (after 8 months of an iron diet).

## 2. Materials and methods

### 2.1. Experimental animal protocols

Male *Hjv*^−/−^ (HJVKO) mice (kindly provided by Dr. Nancy C. Andrews, Duke University) were bred in-house at the University of Alberta Health Sciences Laboratory Animal Services facility. All experiments were performed in accordance with the University of Alberta institutional guidelines, which conformed to guidelines published by the Canadian Council on Animal Care and the Guide for the Care and Use of Laboratory Animals published by the US National Institutes of Health (revised 2011). We performed advanced iron overload protocol by feeding 4 weeks old HJVKO mice with the high iron diet (28) (Prolab®RHM 3000 with iron 380 ppm) until they were 1 year old. At 9 months, the amlodipine group received amlodipine in drinking water (95 mg/L) for 3 months. The vehicle group comprised male HJVKO mice receiving a regular diet without treatment.

### 2.2. Case of human primary overload cardiomyopathy

This patient was consented with written informed consent as part of the Heart Function and Cardiomyopathy Clinic registry, which is approved by the Ethics Committee at the University of Alberta (Pro00077124). Data for this patient's case was obtained through an electronic chart review.

### 2.3. Human explanted hearts

The study was approved by the Ethics Committee at the University of Alberta, and all patients provided written informed consents in accordance with the Declaration of Helsinki (2008) of the World Medical Association. Left ventricular tissue was harvested from explanted human failing hearts and donor non-failing human hearts *via* HELP (Human Explanted Heart Program) at the Mazankowski Alberta Heart Institute and the HOPE (Human Organ Procurement and Exchange) program at the University of Alberta Hospital. The harvested hearts were preserved in cold cardioplegia solution, and collected samples were snap-frozen in liquid nitrogen within 15 min of explantation and stored at −80°C.

### 2.4. Tissue iron levels

Samples (20 mg) of frozen tissue from LV were subjected to inductively coupled plasma resonance mass spectrometry to quantify iron levels at the Trace Metals Laboratory, London, Western Ontario. The samples were analyzed in triplicates, and the average values were used (15, 26).

### 2.5. Scanning electron microscopy

Images were obtained on Hitachi S5500 SEM. Energy-dispersive X-ray spectra were acquired using Oxford INCA Energy in STEM mode with a 1.5 nm probe.

### 2.6. Echocardiography

Transthoracic echocardiography was performed at 1 year of iron overload phenotype mice with the Vevo 2100 high-resolution imaging system equipped with a 30-MHz transducer using 1.5%–2% isoflurane (26, 29).

### 2.7. Hemodynamics

Mice were anesthetized with 1.5% isoflurane, and the right common carotid artery was cannulated. 1.4F Scisense catheter (Scisense Inc.) was advanced through the aortic valve and placed into the LV chamber. Pressure-volume loops were recorded *via* TCP-500 amplifier (Scisense Inc.) and analyzed offline using LabScribe 2 software (IWorks Inc.) as described previously (26, 29).

### 2.8. Measurement of single-myocyte excitability, contractility, and Ca^2+^ transients

Myocytes were enzymatically isolated as described previously (30) without blebbistatin to preserve contractility. After isolation, myocytes were kept in the perfusion buffer solution (pH 7.4). An aliquot of isolated cardiomyocytes was transferred to a bath atop of an inverted microscope to measure excitability, contractility, or Ca^2+^ transients (loaded with FURA-2AM). The measurements were done in myocytes superfused with Tyrode's solution (1.2 mM Ca^2+^) at 35–36°C and paced with field stimulation at 1 Hz as previously described (26, 30, 31). Action potentials were recorded in the whole-cell ruptured-patch configuration with K^+^ pipette solution (31).

### 2.9. In-vivo electrocardiographic (ECG) recording

Mice were placed under isoflurane anesthesia (1.5%–2%) on a heated pad (body temperature maintained at 37°C, measured by the rectal probe). ECG leads were placed in Lead I configuration. The signal was digitized using acquisition interface ACQ-7700 (Data Science International, USA) with P3 Plus software (ver. 5.0, Data Science International, USA).

### 2.10. Histology

The excised hearts from anesthetized mice were arrested in diastole using saline with 15 mM KCl, fixed in 10% buffered formalin, and embedded in paraffin. Thin sections (5 µm) were stained with Prussian blue, picro-sirius red (PSR), Masson trichrome, or wheat germ agglutinin (WGA) stain as described previously (26, 32). Iron depositions were visualized as blue depositions using a bright field view. Myocardial fibrosis was evaluated by quantifying PSR stained sections, and myocyte cross-sectional area (MCSA) was assessed from WGA staining using an Olympus IX81 microscope. Image analysis was done using MetaMorph software (26, 32).

### 2.11. Immunofluorescence

Immunofluorescence was performed on formalin-fixed paraffin-embedded heart sections (5 µm). Briefly, the sections were deparaffinized, followed by antigen retrieval and blocked with blocking buffer (1% BSA in 1X PBS) for 1 h. Similarly, OCT-embedded sections were fixed with 4% paraformaldehyde for 20 min and rehydrated in PBS for 30 min. Then, sections were incubated overnight in a humidified chamber at 4°C with primary antibody against rat anti-mouse neutrophil (Serotec), rat anti-mouse F4/80 (Serotec), mouse anti-nitrotyrosine (Santa Cruz), mouse anti-4-HNE (Abcam). Finally, sections were incubated with different fluorophore-conjugated secondary antibodies (Invitrogen USA), as described previously (26, 32).

### 2.12. Measurement of lipid peroxidation and glutathione levels

The levels of malondialdehyde (MDA), an indicator of lipid peroxidation, were measured in myocardial tissue (100–150 mg) by using a commercially available kit (Bioxytech, MDA-586TM assay, Oxis International Inc., Foster City, CA). Myocardial reduced (GSH) and oxidized glutathione (GSSG) levels were measured as described previously (26, 33).

### 2.13. Taqman real-time PCR

mRNA expression levels were evaluated using TaqMan real-time PCR. Total RNA was extracted from flash-frozen LV tissue using the TRIzol RNA extraction method. RNA (1 µg) was subjected to reverse transcription to synthesize cDNA. Samples were loaded in triplicate, and the data were analyzed by Roche's Light cycler® 480 system.

### 2.14. Bulk RNA sequencing

Transcriptome sequencing, including sample preparation, library construction, and Illumina sequencing, were carried out by Novogene Corporation Inc. (California, USA). The reported methods below were modified based on the standard procedures provided by Novogene. Total RNAs from left ventricles (4 hearts/group) were extracted. The pathway analysis was performed using iDEP.94 http://bioinformatics.sdstate.edu/idep94/ (34).

### 2.15. Western blot analysis

Western blot analysis was performed on flash-frozen LV tissue samples as previously described (26, 32). Briefly, we extracted protein from LV tissues and performed immunoblotting for various proteins using the following primary antibodies: SERCA2a, NCX1 (Thermo Fisher Scientific), PLN-P^Ser16^, total PLN (Badrilla Ltd), Akt-P^Ser473^, Akt-P^Thr308^, total Akt (Cell Signaling), AMPK-P^Thr172^ and total AMPK (Cell Signaling) and subsequently incubated with HRP conjugated secondary antibodies respectively.

### 2.16. Statistical analysis

All data are presented as mean ± SEM. The explanted heart was assessed using the one-sample *t*-test. In the case of multiple group comparisons, differences were evaluated by one-way ANOVA followed by Tukey's post-test. Statistical analysis was performed using Origin 2020 software (OriginLab).

## 3. Results

### 3.1. Amlodipine reduces cardiac iron levels but not hepatic iron levels

**Figure 1:**
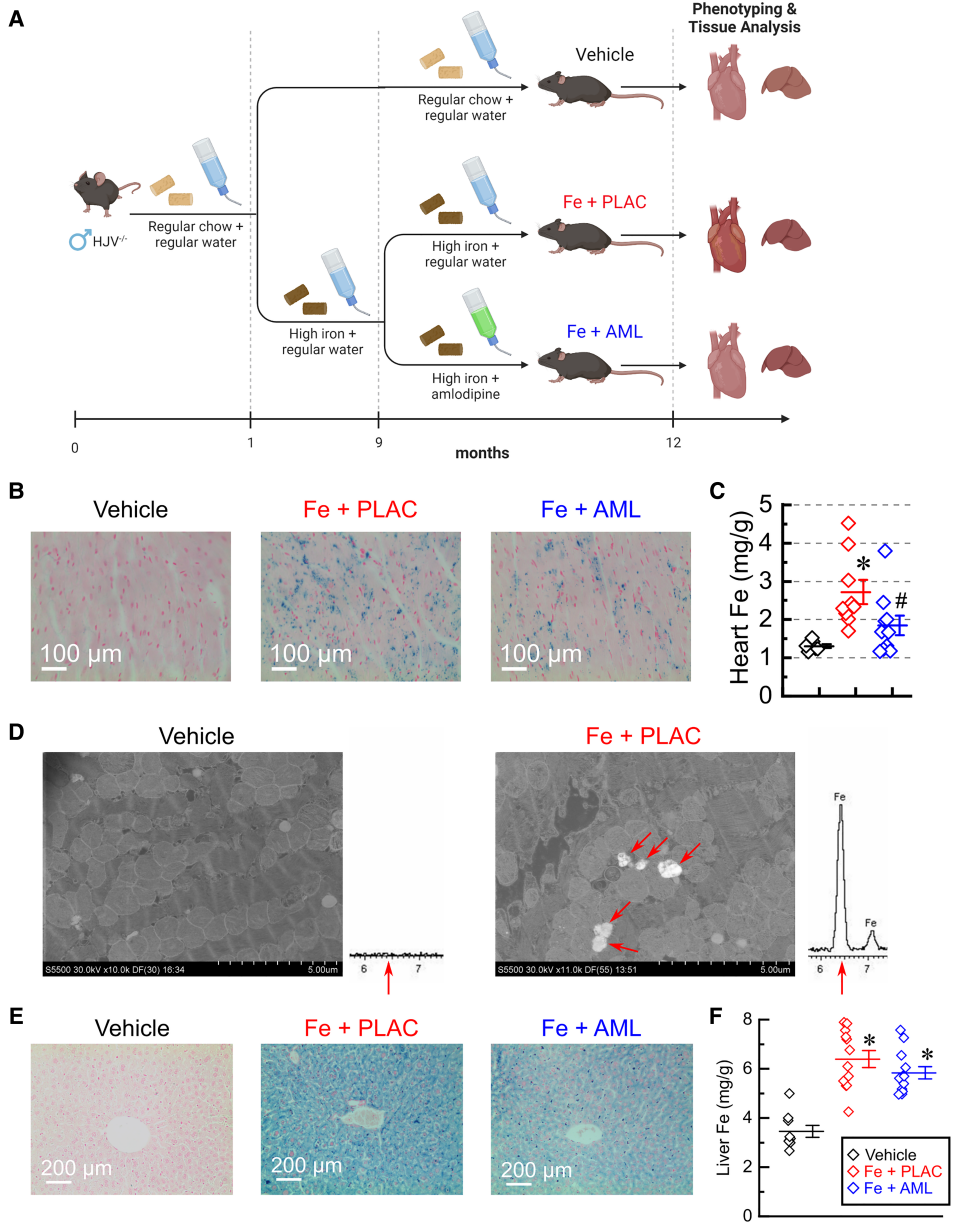
Amlodipine reduces cardiac iron levels. (**A**) The schematic of the study design (created with BioRender.com). (**B**) Representative images of Prussian blue staining. (**C**) Total myocardial iron levels (per dry weight; *n* = 7–10 per group) measured by inductively-coupled plasma mass spectrometry. (**D**) Representative scanning electron microscopy images (iron deposits marked by red arrows) and energy-dispersive X-ray spectra for vehicle and iron groups. Spectrum from the iron group, but not from the vehicle group, has a characteristic Fe peak at 6.4 keV (red arrow). (**E**) Representative images of Prussian Blue staining of liver sections. (**F**) Quantification of iron levels in the liver by inductive plasma mass-spectrometry (*n* = 10–14 per group). ppm = parts per million; *p < 0.05 compared with the vehicle group. ^#^*p* < 0.05 compared with the Iron (Fe) group.

HJVKO mice were fed a high-iron diet to achieve iron overload as mice aged to 12 months. In the rescue group, amlodipine was introduced at 9 months (Figure 1A). Prussian blue histological staining showed a marked increase in iron deposition in groups receiving the high-iron diet (Figure 1B). Direct measurement of iron levels with inductively-coupled plasma mass spectrometry showed that the iron-fed group had markedly elevated iron levels and the amlodipine group had significantly reduced iron levels (Figure 1C). At the cellular level, iron deposition occurred as sub-micrometer particles (Figure 1D). The composition of the deposits was confirmed using energy-dispersive X-ray spectra, which showed a characteristic Fe peak at 6.4 keV (Figure 1D). In contrast to the heart, liver iron levels were increased at 12 months in both iron-fed groups (placebo and amlodipine; see Figures 1E, F). These results demonstrate that amlodipine reduces the iron load in the heart but not in the liver.

### 3.2. Amlodipine improves systolic and diastolic function

#### 3.2.1. Echocardiography

**Figure 2:**
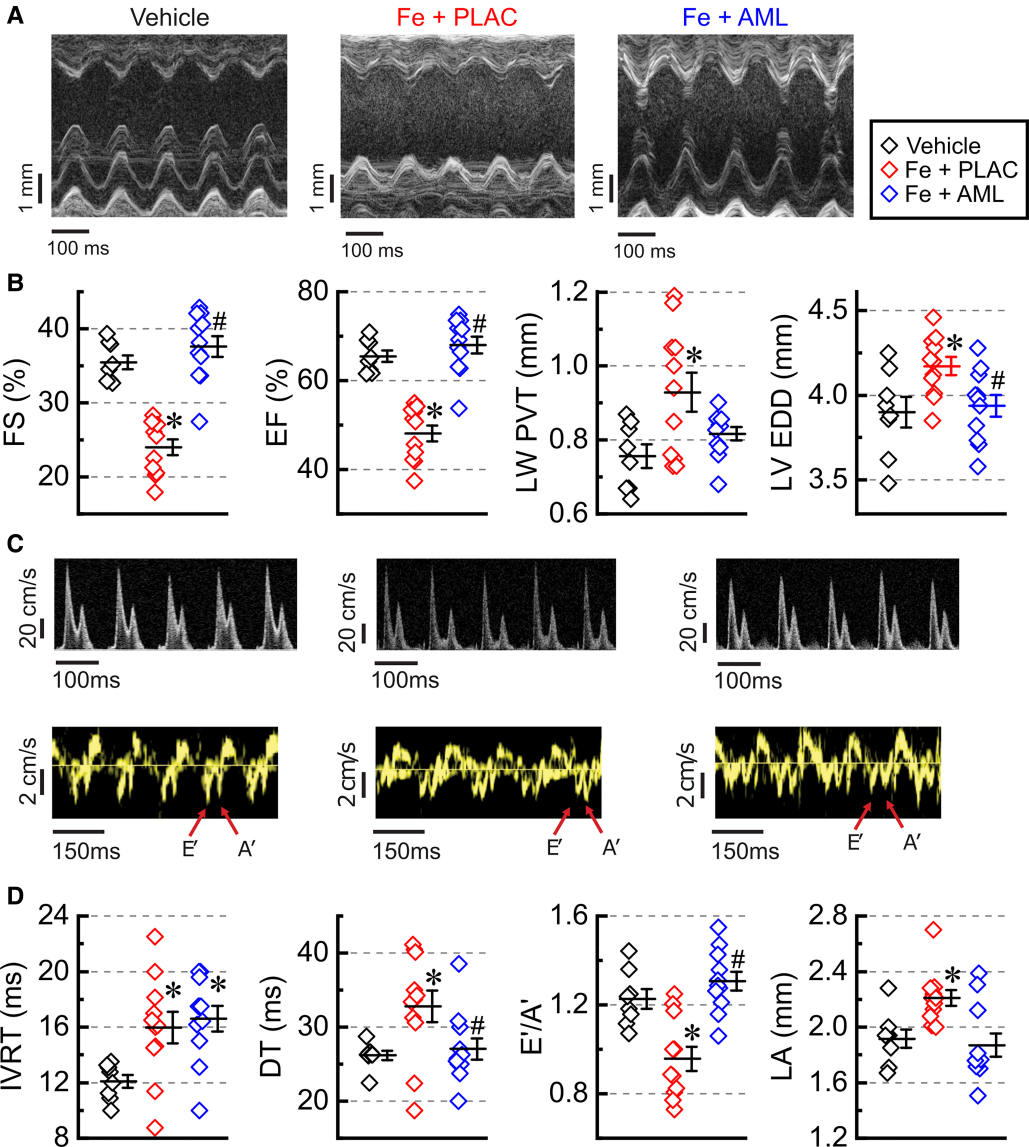
Amlodipine improves systolic and diastolic function. (**A**) Representative M-mode images. (**B**) Quantification of systolic function: fractional shortening (FS), ejection fraction (EF), left ventricular posterior wall thickness (LV PWT), and left ventricular end-diastolic dimensions (LV EDD). (**C**) Representative Doppler (top row) and Tissue Doppler (bottom row) images. (**D**) Quantification of diastolic function: isovolumic relaxation time (IVRT), deceleration time (DT), E prime (*E*′) to A prime (*A*′) ratio (*E*′/*A*′) of Tissue Doppler velocities, and left atrial dimensions (LA). *n* = 8–11 per group. *p < 0.05 compared with the control group; ^#^*p* < 0.05 compared with the iron group.

**Figure 3:**
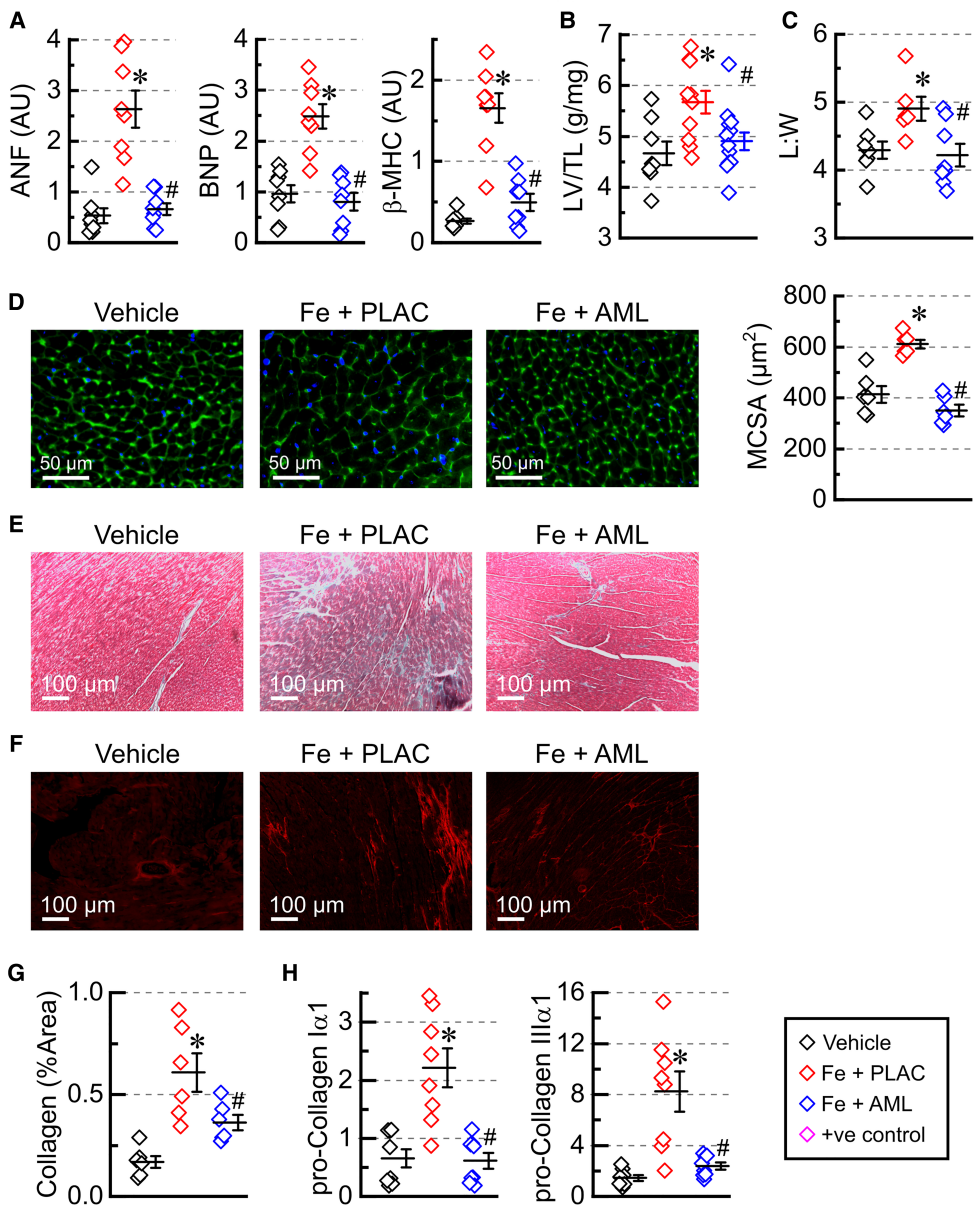
Amlodipine reverses hypertrophic cardiac remodeling. (**A**) Expression levels of heart failure markers: atrial natriuretic factor (ANF), brain natriuretic peptide (BNP), and myosin heavy chain β isoform (β-MHC) (*n* = 8 per group). (**B**) Morphometric index of cardiac hypertrophy (left ventricular weight (LV) adjusted by tibia length (TL); *n* = 8–13). (**C**) Morphometric index of cellular hypertrophy: length to width ratio (*L*:*W*) for isolated myocytes (6–8 hearts per group 50–150 myocytes per heart). (**D**) Representative images of wheat germ agglutinin (WGA) staining and myocytes cross-sectional area (MCSA) measured from WGA staining images (6 hearts per group, 4 sections per heart). (**E**) Representative images of Mason's trichrome staining. (**F**) Representative images of picrosirius-red (PSR) staining and quantification of PSR stained sections (**G**); 6 hearts per group; 4 sections per heart. (**H**) Expression levels of pro-collagens (*n* = 8 hearts per group). *p < 0.05 compared with the control group; ^#^*p* < 0.05 compared with the iron group.

Advanced iron overload led to a significant reduction of systolic function, and this reduction was rescued by treating iron-fed mice with amlodipine (Figure 2A). In response to iron overload, the systolic function was substantially reduced (reductions in fractional shortening, FS, and ejection fraction, EF; Figure 2B), and this reduction was accompanied by LV hypertrophy (increase in LV posterior wall thickness, LV PWT; Figure 2B) and LV dilation (increase in LV end-diastolic dimensions, LV EDD; Figure 3B). Amlodipine treated group showed normalization of systolic function and LV dilation (normalization of FS, EF, and LV EDD; Figure 2B). Noticeable change toward normalization of hypertrophic status in the amlodipine group did not achieve significance (*p* = 0.096; LV PWT, Figure 3B). Mitral and tissue Doppler measurements showed that diastolic function markedly deteriorated in response to iron overload (Figure 2C). Iron overload resulted in impaired cardiac relaxation as evident from (i) an increase in isovolumic relaxation time (IVRT), (ii) an increase in deceleration time (DT), and (iii) a decrease in the ratio of annular velocities (*E*′/*A*′) (Figure 2D). These functional changes were also accompanied by left-atrial (LA) dilation (Figure 2D). The amlodipine-treated group exhibited normalization of all diastolic parameters but one (IVRT) (Figure 2D). In short, iron overload diminished both systolic and diastolic function, whereas amlodipine was able to rescue most of these developments.

#### 3.2.2. Hemodynamics (pressure-volume loop analysis)

Assessment of cardiac function using pressure-volume loop technique showed that (i) iron overload resulted in dilation (increase in LV end-diastolic volume, LV EDV; Table 1), (ii) systolic dysfunction (reductions in ejection fraction, EF; pre-load adjusted contractility, *dP*/*dt*_max_/EDV; and end-systolic pressure-volume relationship, ESPVR; Table 1), and (iii) diastolic dysfunction [elevated LV end-diastolic pressure, LVEDP; and larger isovolumic relaxation time constant, τ (Glantz); but increase in end-diastolic pressure-volume relation, EDPVR, was not statistically significant; Table 1]. The amlodipine-treated group had (i) normalized cardiac volume (LVEDV; Table 1), (ii) normalized systolic function (normal and nearly-normal values for EF, *dP*/*dt*_max_/EDV, and ESPVR), and (iii) partial normalization of diastolic function (some reduction in LVEDP (not significant), normalization of isovolumic relaxation, τ (Glantz), and statistically insignificant reduction in EDPVR). In the case of pressure-volume loops, the results were similar to echocardiography: dilation, systolic and diastolic dysfunctions in the iron group, and quasi-normal cardiac function in the amlodipine-treated group).

**Table 1:** Echocardiographic and hemodynamic (pressure-volume loop) assessment.

	HJVKO Placebo	HJVKO Iron	HJVKO-Iron-CCB
*n*	8	12	8
HR (bpm)	418 ± 8	491 ± 10*	388 ± 11#
LVEDP (mmHg)	2.94 ± 0.52	12.62 ± 2.13*	8.60 ± 1.90
LVESP (mmHg)	120 ± 4.39	135 ± 3.84*	117.3 ± 4.58#
LVEDV(µl)	29.5 ± 2.2	43.85 ± 4.0*	30.2 ± 4.2#
LVESV (µl)	6.45 ± 0.9	20.26 ± 4.77*	4.77 ± 2.24#
SV(µl)	23.07 ± 1.62	23.59 ± 2.28	25.47 ± 2.9
EF (%)	79.55 ± 1.79	59.20 ± 6.74*	85.81 ± 4.6#
*dP*/*dt*_max_/EDV (mmHg/s/µl)	343.2 ± 23	204.5 ± 27.2*	299 ± 25.3#
ESPVR (mmHg/µl)	5.58 ± 0.6	3.19 ± 0.5*	5.85 ± 1.7#
τ (Glantz) (ms)	12.3 ± 0.7	21.13 ± 2.4*	14.3 ± 0.2#
EDPVR (mmHg/µl)	0.117 ± 0.017	0.135 ± 0.016	0.105 ± 0.025

HR, heart rate; LVEDP, end diastolic pressure; LVESP, end systolic pressure; LVEDV, end diastolic volume; LVESV, end systolic volume; SV, stroke volume; EF, ejection fraction; *dP*/*dt*_max_/EDV, Starling's contractile index; ESPVR, end-systolic pressure-volume relationship; Tau (τ), LV relaxation time constant; EDPVR, end-diastolic pressure-volume relationship.

*p < 0.05 compared with the placebo; ^#^*p* < 0.05 compared with the iron group.

### 3.3. Amlodipine reverses hypertrophic cardiac remodeling

Iron accumulation led to elevated expression levels of heart failure markers (natriuretic peptides, ANF and BNP, and myosin heavy chain β, β-MHC) in the iron group. In contrast, the amlodipine-treated group exhibited overall normalization of expression of heart failure markers (Figure 3A). In addition, the morphometric index of hypertrophy, LV mass adjusted by tibia length (LV/TL), was increased in the iron group but normalized in the amlodipine-treated group treated with amlodipine (Figure 3B). At the cellular level, measurement of length (*L*) and width (*W*) of isolated cardiomyocytes demonstrated that myocytes were elongated (increased *L*:*W* ratio) in the iron group and had an *L*:*W* ratio close to normal in the amlodipine-treated group (Figure 3C). Assessment of hypertrophy at the tissue level using WGA staining (Figure 3D) revealed increased MCSA in the iron group and substantially reduced myocyte area in the amlodipine-treated group. The data suggest that iron overload leads to eccentric-hypertrophy remodeling, and amlodipine treatment reversed a significant part of hypertrophic remodeling.

**Figure 4:**
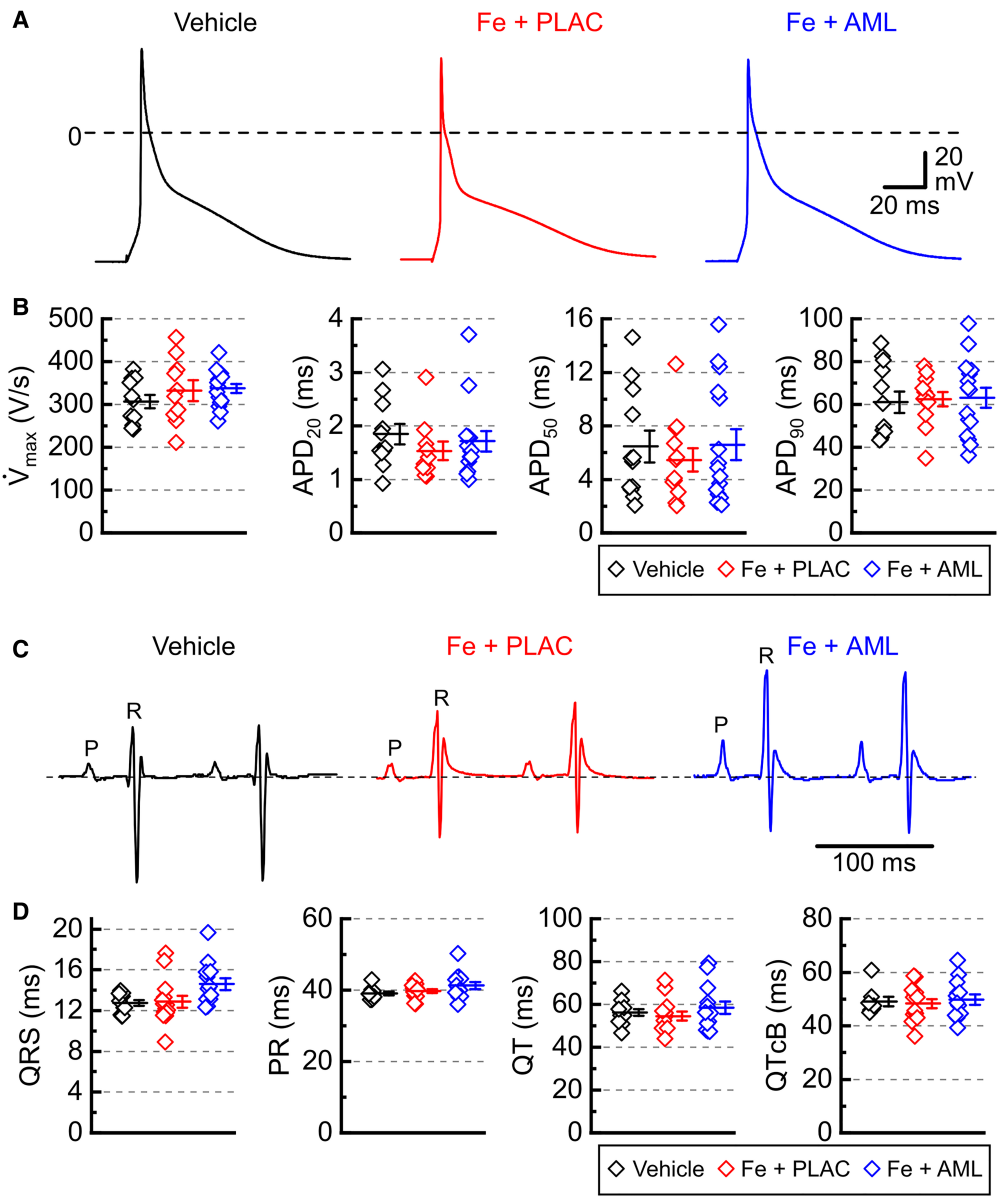
Cardiac electrical activity in murine iron overload. (**A**) Representative action potentials for the vehicle, iron with placebo (Fe + PLAC), and iron with amlodipine (Fe + AML) groups (left to right). (**B**) Maximal rate of rise of action potential (V̇_max_) and action potential durations for 20, 50, and 90% of repolarization (APD_20_, APD_50_, and APD_90_, respectively); 3 hearts /group; 4–6 cells /heart. (**C**) Representative electrocardiograms for the vehicle, iron with placebo (Fe + PLAC), and iron with amlodipine (Fe + AML) groups. (**D**) Durations of QRS, PR, QT, and QT Bazett corrected (QTcB) intervals (*n* = 11–14 per group).

### 3.4. Amlodipine reduces cardiac fibrosis

Cardiac fibrosis was assessed using Mason's trichrome (Figure 3E) and PSR (Figure 3F) staining. The quantification of PSR staining (Figure 3G) showed a marked increase in interstitial fibrosis in iron-overloaded hearts and a considerable reduction of fibrosis in the hearts from the amlodipine-treated group. Expression levels of pro-fibrotic markers (pro-collagen Iα1 and IIIα1) followed a similar pattern: upregulation in the iron overload group and normalization in the amlodipine-treated group (Figure 3H).

### 3.5. Amlodipine normalizes single-myocyte contractility and Ca^2+^ cycling

#### 3.5.1. Excitability

Measurements of electrical activity (action potentials) in the isolated cardiac myocytes showed no significant change in the major parameters of action potential: the maximal slope of depolarization (*V̇*_max_) and action potential duration (APDs corresponding to repolarization levels of 20%, 50%, and 90%; Figures 4A, B) suggesting that iron and amlodipine effects on contractility were driven primarily *via* changes in Ca^2+^ cycling. The lack of the effect of iron and amlodipine on cardiac electrical activity was corroborated by electrocardiographic measurements. The Lead I electrocardiogram in anesthetized mice revealed no substantial differences in ECG parameters between the groups (Figures 4C, D).

#### 3.5.2. Contractility

**Figure 5:**
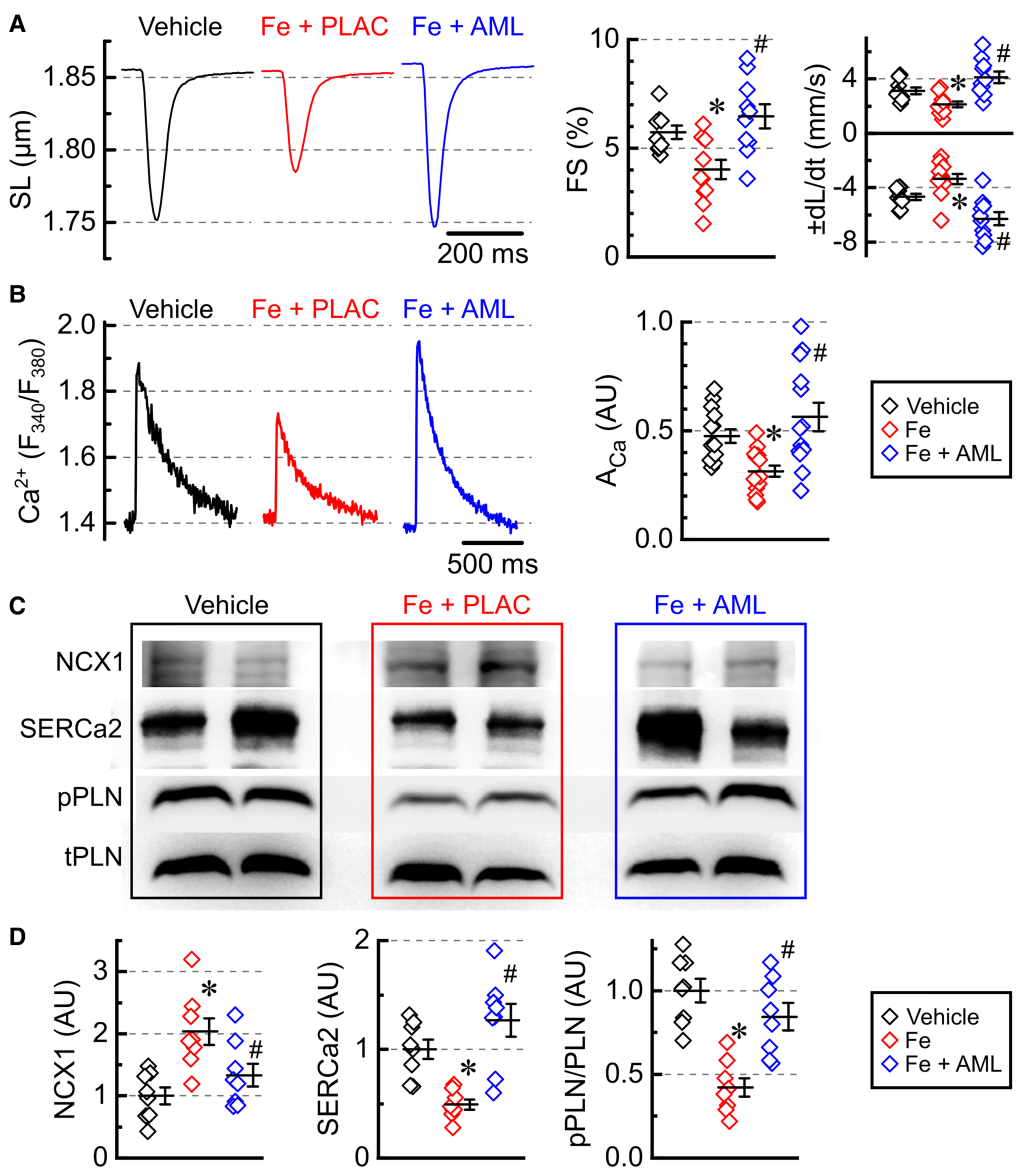
Amlodipine normalizes single-myocyte contractility and Ca^2+^ cycling. (**A**) Averaged recording of sarcomeric length (SL), fractional shortening (FS), contractility (−*dL*/*dt*), and relaxation (+*dL*/*dt*) (2 hearts per group 4–6 cells per heart). (**B**) Averaged recording of Ca^2+^ release transients plotted as the ratio of FURA-2 fluorescence (F_380_/F_340_) and Ca^2+^ release amplitudes (*A*_Ca_). (**C**) Representative western blots for Na^+^–Ca^2+^ exchanger (NCX1), sarcoplasmic reticulum Ca^2+^ ATPase (SERCa2), Ser16-phosphorylated phospholamban (pPLN), and total phospholamban (tPLN). (**D**) Protein levels for NCX1 and SERCa2 and phosphorylation ratio for phospholamban (pPLN/tPLN), quantified from western blots (*n* = 8 hearts per group). *p < 0.05 compared with the control group; ^#^*p* < 0.05 compared with the iron group.

Single-myocyte contractility was inhibited in the myocytes isolated from iron-overloaded hearts (Figure 5A). Both contractility (FS and maximal velocity of contraction, −*dL*/*dt*) and relaxation (maximal velocity of relaxation, +*dL*/*dt*) were diminished in the iron group (Figure 5A). Conversely, the amlodipine-treated group showed normalization of both contractility and relaxation parameters (Figure 5A).

#### 3.5.3. Ca^2+^ cycling

The amplitude of Ca^2+^ transients (*A*_Ca_) was inhibited in the iron-overload group and normalized in the amlodipine-treated group (Figure 5B). To investigate what underlines changes in Ca^2+^ transients, we assessed levels and phosphorylation state of three major proteins that control the time constant and amplitude of Ca^2+^ transient: Na^+^–Ca^2+^ exchanger (NCX1), sarco-endoplasmic reticulum Ca^2+^-ATPase (SERCa2), and phospholamban (PLN) (Figure 5C). NCX1 levels were markedly elevated in the iron-overload group and mostly normalized in the amlodipine-treated group (Figure 5D). SERCa2 levels were lowered in the iron-overload group, but roughly normalized in the amlodipine-treated group (Figure 5D), which is consistent with the normalization of Ca^2+^ transient amplitude (*A*_Ca_) (Figure 5B). PLN phosphorylation was decreased in the iron group and was normalized in the amlodipine-treated groups (Figure 5D).

### 3.6. Amlodipine normalizes oxidative stress and metabolic signaling

**Figure 6:**
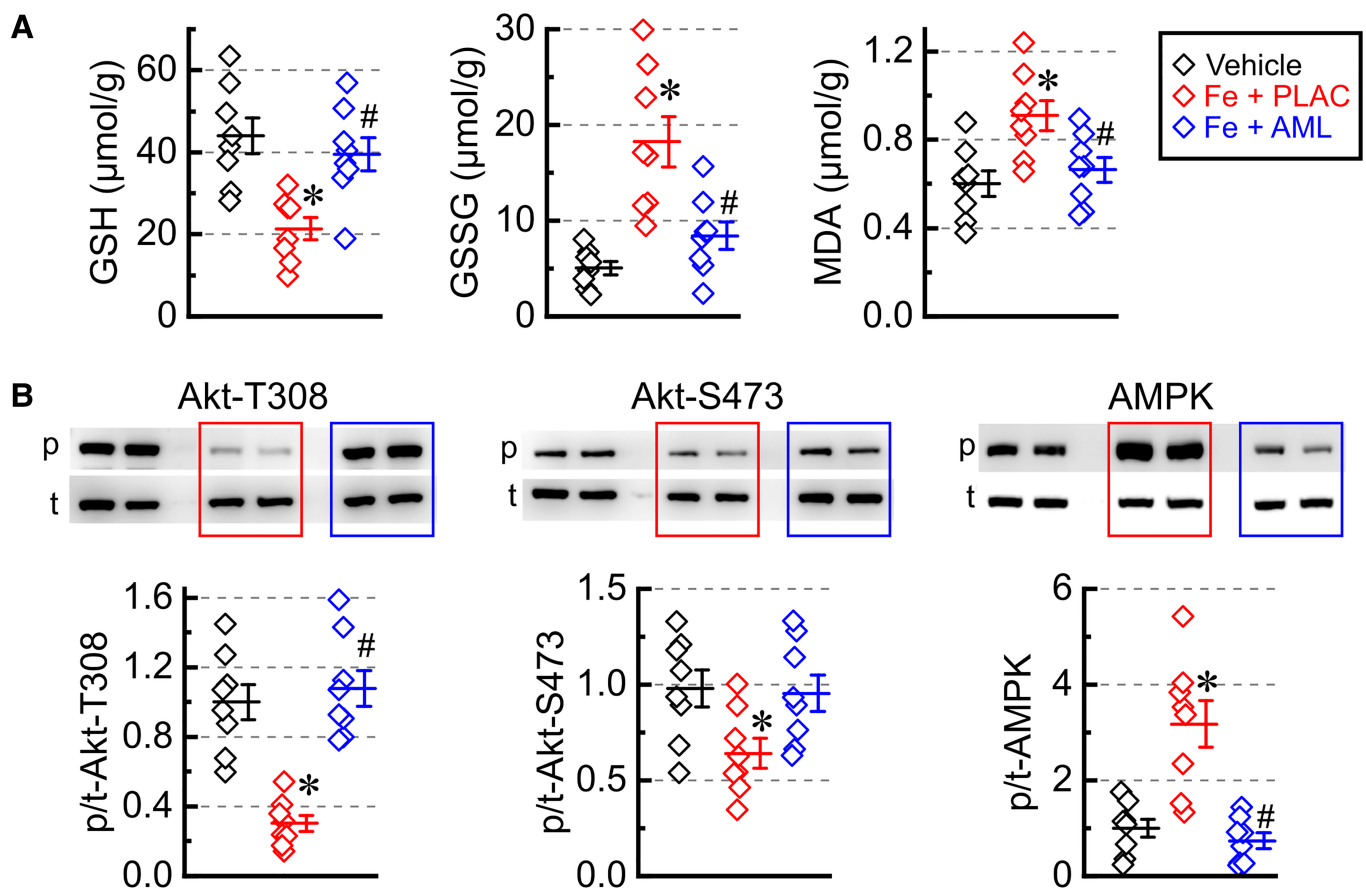
Amlodipine normalizes oxidative stresses and metabolic signaling. (**A)** Concentrations of reduced glutathione (GSH), oxidized glutathione (GSSG), and malondialdehyde (MDA) (*n* = 8 per group). (**B**) Phosphorylation levels (*p*/*t*) for Akt [Thr308 phosphorylation site (Akt-T308, *p*), Ser473 phosphorylation site (Akt-S473, *p*), and total Akt (*t*)] as well as AMP-activated protein kinase [AMPK; Thr172 phosphorylation site (*p*) and total (*t*)]. *p < 0.05 compared with the control group; ^#^*p* < 0.05 compared with the iron group.

The state of the intrinsic antioxidant system was assessed by measuring the concentration of GSH and GSSG. Consistent with a heightened degree of oxidative stress, the iron group had low GSH and high GSSG concentrations (Figure 6A); on the other hand, the amlodipine-treated group had a close-to-normal pattern of GSH-GSSG distribution (high GSH with low GSSG) (Figure 6A). MDA was elevated in the iron group but largely normalized levels in the amlodipine-treated group, corroborating GSH-GSSG results that oxidative stress was elevated in the iron group and was at near-normal levels in the amlodipine-treated hearts (Figure 6A). Assessment of metabolic signaling showed that iron overload was associated with (i) decreased Akt phosphorylation at both Thr308 (Akt-T308) and Ser473 (Akt-S473) and (ii) increased AMPK phosphorylation (Thr172) (Figure 6B). In contrast, the amlodipine-treated group had near-normal phosphorylation levels at all three sites (Figure 6B), suggesting that the amlodipine treatment normalizes pathological metabolic signaling in cardiac tissue.

**Figure 7:**
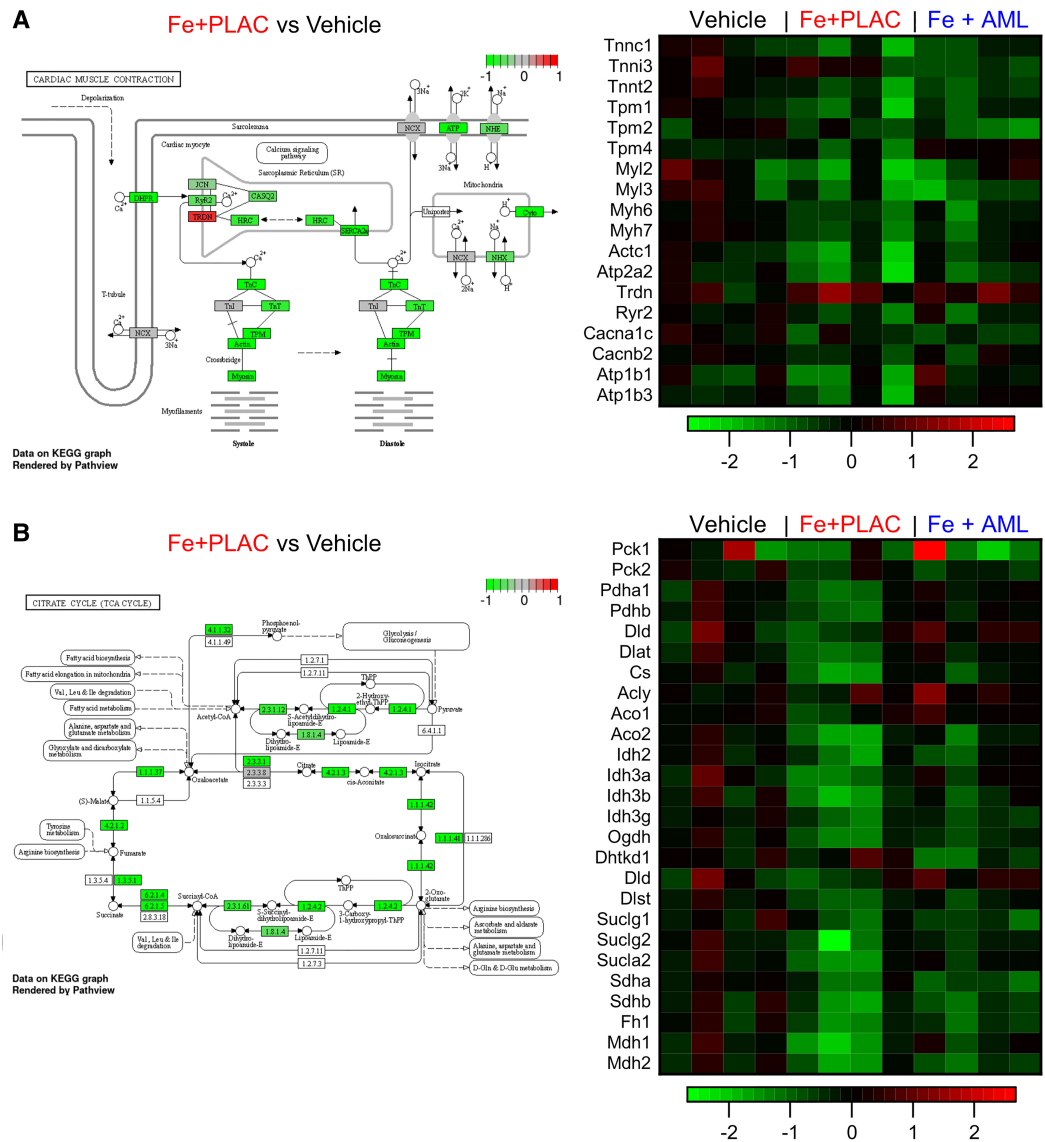
Iron overload reduces RNA expression of proteins in the cardiac muscle contraction pathway and citrate cycle. (**A**) Relative expression changes in cardiac muscle pathway and heat map of relative expression changes of selected RNA from cardiac muscle pathway. (**B**) Relative expression changes in citrate cycle and heat map of relative expression changes of selected RNA from citrate cycle.

Iron overload alters the expression of multiple genes involved in cardiac muscle contraction and the citrate cycle. We used bulk RNA sequencing to assess broad changes in expression levels (Data Supplement and Supplementary Figure S1) and found two important pathways being affected: cardiac muscle contraction and citrate cycle. In both pathways, iron overload led to an overall reduction in the expression levels (cardiac muscle contraction pathway Figure 7A and citrate cycle Figure 7B). Amlodipine therapy partially normalized the changes in myocardial gene expression in response to iron overload (Figures 7A, B).

### 3.7. Adverse remodeling in an explanted human heart with IOC

A 16-year-old girl with β-thalassemia major was admitted with refractory heart failure (HF). Echocardiogram showed dilation with bi-ventricular systolic and diastolic dysfunction with LVEF of 25%. The pre-transplant iron panel showed elevated serum iron (31 µmol/L; normal: 9.0–30 µmol/L), severely high transferrin iron saturation (94%; normal: 25%–35%) and low hemoglobin (94 g/L; normal: 120–160 g/L). Pre-transplant cardiac MRI indicated severe iron overload. With proper consent, samples were collected from the explanted heart for further comparison to non-failing control heart samples obtained from HOPE.

**Figure 8:**
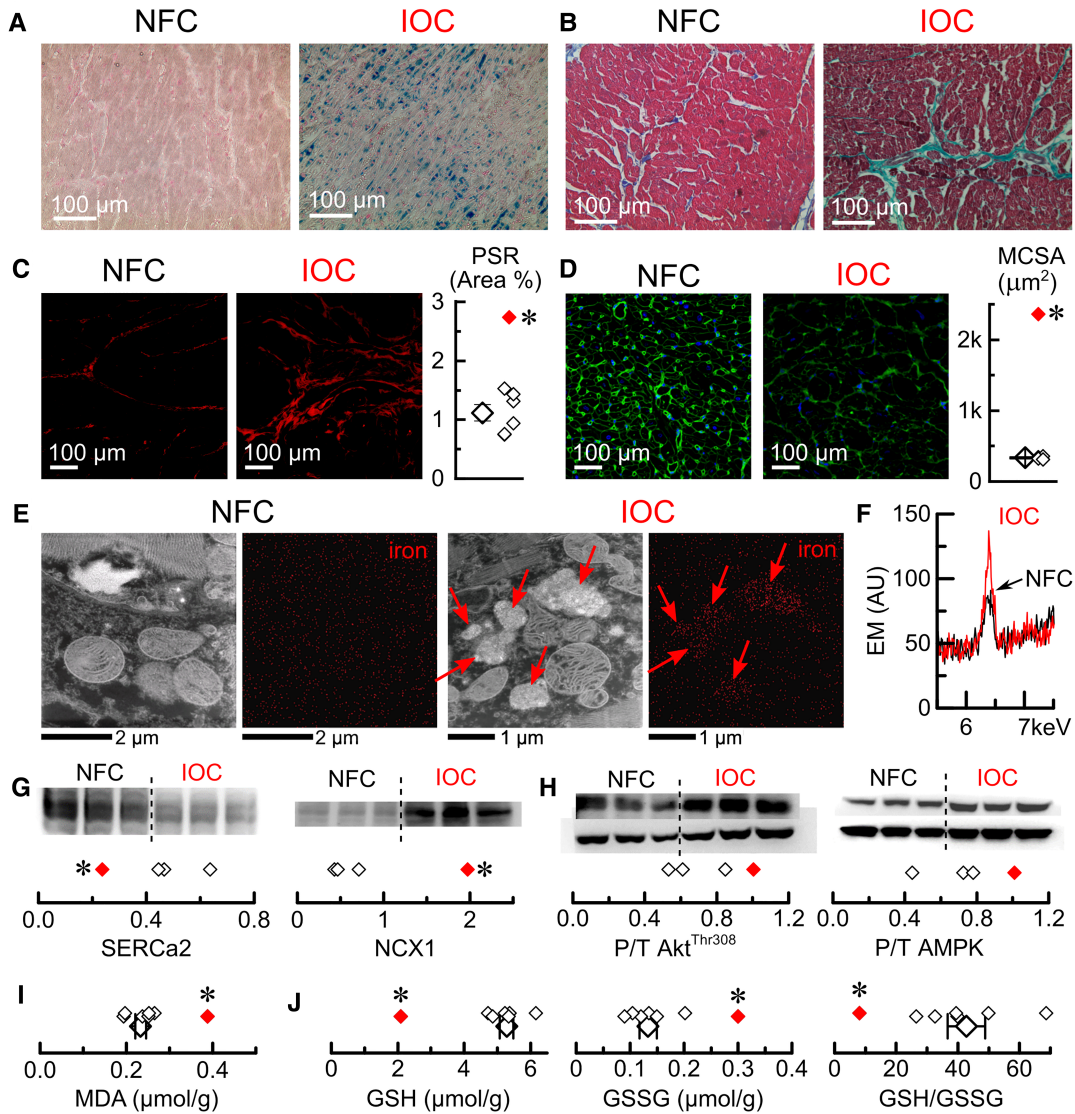
Characterization of the explanted human heart with IOC. (**A**) Representative images of Prussian blue staining. (**B**) Representative images of Mason's trichrome staining. (**C**) Representative images of picrosirius-red (PSR) staining and quantification of PSR stained sections. (**D**) Representative images of wheat germ agglutinin (WGA) staining and myocytes cross-sectional area (MCSA) measured from WGA staining images. (**E**) Representative scanning-electron microscopy images for control and iron (Fe) groups (iron deposits marked by red arrows). (**F**) Superimposed energy-dispersive X-ray spectra for non-failing controls (Ctl, black) and iron overload cardiomyopathy (IOC, red), showing the characteristic Fe peak at 6.4 keV. (**G**) Western blots and their quantifications (3 non-failing controls vs. an average of IOC) for sarcoplasmic reticulum Ca^2+^ ATPase (SERCa2) and Na^+^–Ca^2+^ exchanger (NCX1). (**H**) Western blots and their quantifications (3 non-failing controls vs. an average of IOC) for phosphorylation levels of Akt (Thr308 phosphorylation site, Akt^Thr308^) and AMP-activated protein kinase (Thr172 phosphorylation site, AMPK). (**I**) Malondialdehyde (MDA) concentration. (**J**) Reduced glutathione (GSH), oxidized glutathione (GSSG) concentrations, and GSH/GSSG ratio; open symbols—non-failing controls, filled symbol—explanted heart with IOC; *p < 0.05 compared with the non-failing controls.

Prussian blue staining showed widespread myocardial iron deposition in the IOC heart but no detectable staining in the non-failing control hearts (Figure 8A). The IOC heart had marked fibrosis, as evident from trichrome staining (Figure 8B) and PSR staining (Figure 8C), in association with marked hypertrophy judging by the increase in MCSA (Figure 8D). Scanning electron microscopy revealed micron-sized iron deposits in the IOC heart but not in non-failing control (Figure 8E), with spectral analysis showing a marked increase of characteristic Fe peak at 6.4 keV (Figure 8F). Assessment of key proteins involved in Ca^2+^ cycling showed a reduction in the levels of SERCA2 and a compensatory increase in the levels of NCX1 (Figure 8G). Iron overload was also associated with metabolic remodeling, as evidenced by increased phosphorylation ratios of Akt^Thr308^ and AMPK (Figure 8H). Assessment of oxidative stress status showed a marked increase in MDA (Figure 8I), reduction in GSH, increase in GSSG, and lower GSH/GSSG ratio (Figure 8J). In this clinical case, IOC exhibited iron deposition (including intracellular micrometer-size particles), fibrosis, hypertrophy, remodeling of Ca^2+^ cycling, metabolic remodeling, and a heightened degree of oxidative stress compared to non-failing control hearts. One-year post-cardiac transplant, this patient is on chelators (deferiprone and deferoxamine) and L-type Ca^2+^ channel blocker (amlodipine), has no signs of heart failure, and is followed up regularly. Cardiac MRI showed no evidence of myocardial (T2* = 45.5 ms) or hepatic (T2* = 17.5 ms) iron overload and normal bi-ventricular size and function, with LVEF of 66% and normal right ventricular ejection fraction of 63%.

### 3.8. Amlodipine rescues a patient with IOC due to primary hemochromatosis

**Figure 9:**
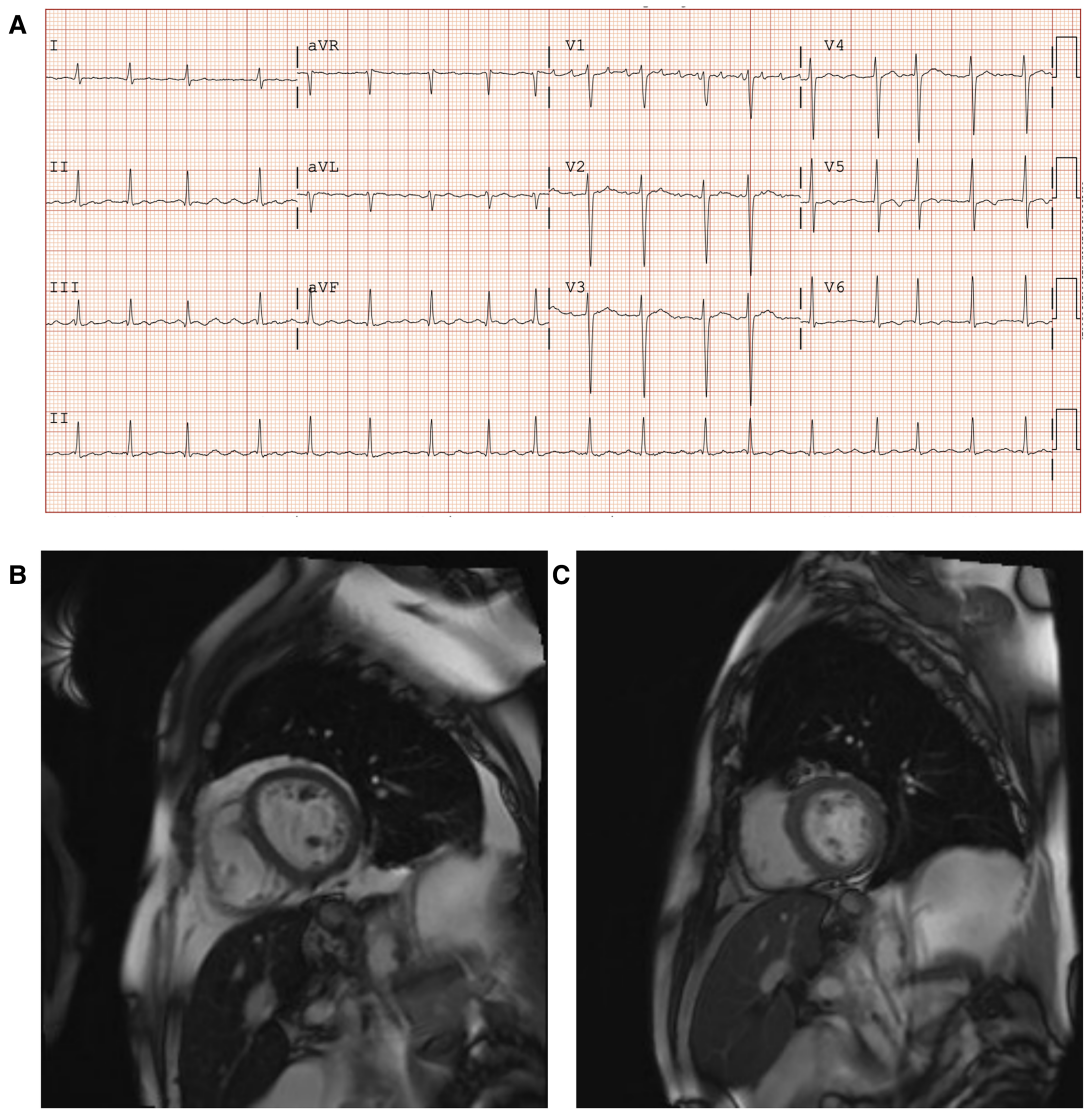
Patient with primary hemochromatosis and IOC treated with amlodipine. (**A**) Twelve-lead electrocardiogram at initial clinic visit demonstrating irregular conduction with an atrial flutter at 105 beats per minute and non-specific lateral t-wave inversion. (**B**) Cardiac magnetic resonance imaging (CMR) with T2 mapping at baseline (October 2019), prior to initiation of amlodipine to iron chelation therapy. T2 mapping showed moderate iron loading of the myocardium (T2* 12–13 ms), and hepatic iron overload was also noted (T2* 2 ms). (**C**) CMR with T2 mapping after iron chelation therapy with adjunctive amlodipine (12 months after baseline CMR, October 2020). Improvement of myocardial T2* to 26 ms, indicating the absence of myocardial iron loading and hepatic T2* improved to 5 ms, indicating mild hepatic iron loading.

A 69-year-old man with a history of primary hemochromatosis (PH) (C282Y/H63D compound heterozygote) and myelodysplastic syndrome (MDS) presented with IOC leading to HF and conduction abnormalities. He reported fatigue, shortness of breath, paroxysmal nocturnal dyspnea, and bilateral leg swelling [New York Heart Association (NYHA) Class III]. Blood work demonstrated anemia (hemoglobin = 87 g/L), elevated serum ferritin (1,230 µg/L), and elevated B-type natriuretic peptide (1,224 ng/L). Electrocardiogram showed abnormal conduction delays with underlying atrial flutter (Figure 9A). Cardiac magnetic resonance imaging (CMR) demonstrated dilated left ventricle with moderately reduced systolic function (LVEF = 40%). Cardiac T2 mapping showed evidence of moderate myocardial iron loading (T2* 12–13 ms) and hepatic iron overload (T2* 2 ms) (Figure 9B). Medical therapies were initiated to manage this patient's IOC and subsequent HF. Given this patient's concurrent MDS and PH, therapeutic phlebotomy was not feasible. Oral iron chelation therapy was undertaken with increasing doses of deferasirox; however, iron saturation remained elevated at 70%. Despite improvements, this patient still expressed marked limitations in physical activities (NYHA class III). He was started on amlodipine 2.5 mg orally daily in addition to iron chelation and was titrated to the maximum dose of 10 mg daily. During follow-up 2 years after adding amlodipine, the patient reported marked improvement in HF symptoms (NYHA class I). Comparing initial CMR imaging to 24 months demonstrated improvement in LVEF and RVEF from 40% to 66% and 41% to 64%, respectively, with normalized ventricular volumes and mass. Myocardial T2* improved from 12.5 ms to 30 ms, indicating a marked reduction in myocardial iron loading (Figure 9C).

## 4. Discussion

Iron homeostasis is essential to maintain cardiac function, and dysfunction of iron metabolism has been implicated in various cardiovascular diseases (35–38). IOC is a major cause of heart failure in patients with hemochromatosis (iron overload) (12, 39–41). Phlebotomy and chelation therapy are the two main approaches to treating and managing iron overload (4, 6, 12, 39–41). However, phlebotomy cannot be used in patients with significant anemia, malignancy, or hemodynamic instability, and chelation therapy has an inherent risk of toxicity (4, 40, 41). Chelation therapies lower overall systemic iron, and amlodipine complements that by hindering the entry of Fe^2+^ *via* L-type Ca^2+^ channels, which are abundant in the heart. Therefore, using amlodipine as an adjunct therapy can improve the effectiveness of chelation therapy (19), creating an opportunity to lower cardiac iron levels faster and with a lower dosage of chelating agents, potentially improving the safety of the therapy.

### 4.1. Characterization of IOC in the explanted human heart and the animal model

Hemojuvelin-deficient mice on an iron-rich diet are an established IOC model that exhibits both systolic and diastolic dysfunction (26, 27). Iron levels obtained in the hearts of the animal model (2.7 mg/g dry weight) were approaching myocardial iron levels (3.5–9.2 mg/g dry weight) reported in patients with IOC and associated heart failure. The presented animal model replicated many crucial changes that develop during IOC in patients. We observed deterioration of cardiac function and micron-sized iron particles in cardiac tissue both in the explanted human heart with IOC and in the mouse model. In the case of the explanted heart, the heart failure was very severe (LVEF = 25%), whereas mice on the iron-rich diet had moderate cardiomyopathy (LVEF = 48%). Despite these few differences, both the explanted heart and the mouse model had fibrosis, hypertrophy, remodeling of Ca^2+^ cycling (decrease in SERCa2 levels accompanied by a compensatory increase in NCX levels), metabolic remodeling (inhibition of Akt activity coupled with upregulation of AMPK activity), and oxidative stress (increased MDA levels and low GSH/GSSG ratio). Accumulation of Fe^2+^ inside the cardiac cells is known to lead to the excessive production of ROS and trigger cell death due to ferroptosis (42), resulting in heart failure (35). In this animal model of IOC, we observed no changes in cardiac excitability but impaired cardiac cellular contractility and relaxation. The diminished contractility was associated with the reduction in Ca^2+^ release suggesting that changes in Ca^2+^ cycling underly reduced cardiac contractility.

### 4.2. Rescuing IOC with amlodipine in the animal model

As an L-type Ca^2+^ channel blocker, amlodipine can reduce the entry of non-transferrin-bound iron (Fe^2+^) *via* L-type Ca^2+^ channels, resulting in lower levels of cardiac iron and reduced oxidative stress in cardiac tissue. Mitigated damage from oxidative stress will contribute to the normalization of expression of Ca^2+^ cycling proteins and collagens, leading to the reversion of fibrosis and normalization of Ca^2+^ cycling, which translates to the normalization of systolic and diastolic dysfunctions at the whole-heart level. Besides being a channel blocker, amlodipine has been shown to have highly efficient antioxidant properties due to high lipophilicity and the ability to quench free radical formation (21). Antioxidants have been shown to mitigate damage associated with iron toxicity. For example, resveratrol (26, 27), thrombopoetin (43), and ebselen (44) have been shown to protect the heart in the settings of iron overload in mouse models. In our study, amlodipine demonstrated properties that can also be attributed to antioxidant activity (normalization of GSH, GSSG, and MDA levels).

### 4.3. Amlodipine as a treatment for IOC

In the reported case of primary hemochromatosis and IOC, the myelodysplastic syndrome prevented therapeutic phlebotomy. Iron chelation alone (deferasirox) was insufficient to reduce the burden of cardiac iron overload. However, adjunctive amlodipine therapy provided objective improvement in cardiac iron overload on imaging and symptomatic improvement in functional status for this patient. Despite randomized clinical trials demonstrating that amlodipine did not exhibit favorable clinical effects for the treatment of chronic heart failure (45, 46), amlodipine therapy could be used as adjunctive therapy in cases of iron overload, as seen in our patient's case and recent randomized clinical trials of amlodipine in patients with iron overload (17, 19). Therefore, any beneficial action from reducing iron deposition in the heart may also be reinforced by the antioxidant properties of amlodipine, suggesting that amlodipine is a good option for an adjuvant agent in chelation therapy.

## 5. Conclusions

We used a genetic model of primary hemochromatosis, human explanted heart tissue with iron overload and a patient with primary hemochromatosis to reveal convergent pathways of iron-mediated injury and heart disease. IOC had a similar pathogenic profile (iron accumulation pattern, histological changes, regulatory protein alterations and ROS status) in the animal model and the explanted human heart. Amlodipine was shown to rescue IOC in the animal model and the reported clinical case of primary hemochromatosis. Amlodipine's ability to suppress Fe^2+^ entry and effectively quench the free radical formation and oxidative damage makes it an effective agent to improve the effectiveness of chelation therapy. The clinical significance of our findings supports the use of amlodipine as an adjuvant therapy in patients with IOC.

## Data Availability

The datasets presented in this study can be found in online repositories. The names of the repository/repositories and accession number(s) can be found below: https://www.ncbi.nlm.nih.gov/sra/PRJNA922223.
