# Supplementary material for: Amlodipine rescues advanced iron overload cardiomyopathy in hemojuvelin knockout murine model: Clinical implications
Source: Front Cardiovasc Med. 2023 Apr 21;10:1129349. doi: 10.3389/fcvm.2023.1129349 (PMC10160373; doi:10.3389/fcvm.2023.1129349)
Supplement: Supplementary file 2 [file Image1.pdf]

Supplementary Material

Amlodipine rescues advanced iron overload cardiomyopathy in hemojuvelin knock-out murine model: clinical implications

Pavel Zhabyeyev[1,2], Chandu Sadasivan[1,2], Saumya Shah[1,2], Faqi Wang[1], and Gavin Y. Oudit[1,2]

[1]Division of Cardiology, Department of Medicine, [2]Mazankowski Alberta Heart Institute, University of Alberta, Edmonton, Canada

*** Correspondence:**
Gavin Y. Oudit, MD, Ph.D., FRCPC
Division of Cardiology, Department of Medicine
Mazankowski Alberta Heart Institute, University of Alberta
8440 112 Street NW, Edmonton,
Alberta, Canada, T6G 2B7
Phone: 780 407 8569
Fax: 780 407 6452
Email: gavin.oudit@ualberta.ca

1 Supplementary Figure

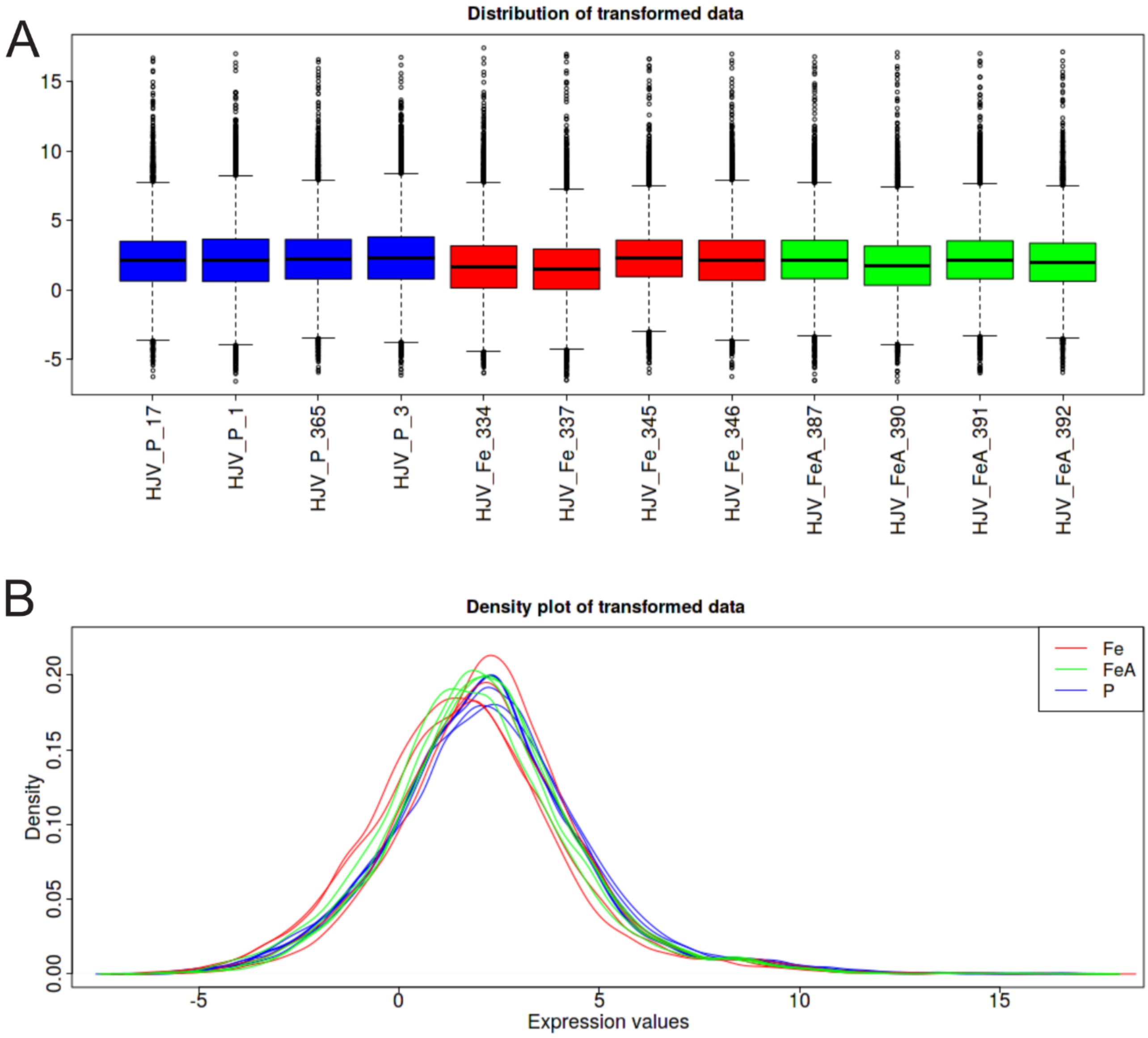

Figure S1. Distribution and reads of all sequenced samples. **A**. Distribution of reads for each samples. **B**. Density distributions for all sequenced samples: vehicle (P), iron (Fe), and iron with amlodipine (FeA).
